# A Comparison of Fresh Pork Colour Measurements by Using Four Commercial Handheld Devices

**DOI:** 10.3390/foods10112515

**Published:** 2021-10-20

**Authors:** Xinyi Wei, Stephanie Lam, Benjamin M. Bohrer, Bethany Uttaro, Oscar López-Campos, Nuria Prieto, Ivy L. Larsen, Manuel Juárez

**Affiliations:** 1 Lacombe Research and Development Centre, Agriculture and Agri-Food Canada, Lacombe, AB T4L 1W1, Canada; xinyi.wei@agr.gc.ca (X.W.); stephanie.lam@agr.gc.ca (S.L.); bethany.uttaro@agr.gc.ca (B.U.); oscar.lopezcampos@agr.gc.ca (O.L.-C.); nuria.prietobenavides@agr.gc.ca (N.P.); ivy.larsen@agr.gc.ca (I.L.L.); 2 Department of Animal Sciences, The Ohio State University, Columbus, OH 43210, USA; bohrer.13@osu.edu

**Keywords:** CIE L*a*b*, Minolta, Nix, Spectro 1, RSD, correlation

## Abstract

The objective of this study was to evaluate the performance of different low-cost instruments to measure pork colour in comparison to Minolta spectrophotometers and industry subjective standards. Canadian pork colour standards and commercial meat (252 loin chops and 46 tenderloins) were measured using two Minolta (CM 700D) spectrophotometers, four Nix sensors (two Nix Pro II and two Nix QC), and four Spectro devices (two Spectro 1 and two Spectro 1 Pro). Using Bland-Altman plots, all hand-held devices revealed similar performance on colour coordinates, except for the Nix Pro II, which had more variability on a* value, and Spectro 1 Pro on b* value, when compared to Minolta measurements. Low RSD values (< 5%) were obtained from repeated measurements on Canadian colour standards. The trend of colour coordinates on colour scores (0–6) were similar for all four commercial instruments, except for a* from Nix Pro and b* from Spectro 1. The correlation coefficients between subjective standards and colour coordinates from the Nix and Spectro devices were slightly higher than the Minolta spectrophotometers. Even though Nix and Spectro 1 series instruments generated different absolute colour coordinate values on meat samples, these pocket-size instruments presented great reliability to measure pork surface colour. However, operational limitations of the instruments, such as the internal calibration time between samples for the Spectro 1 series, should also be considered.

## 1. Introduction

The consumer’s first perception of pork quality is heavily influenced by lean colour, with darker lean colour being more desirable [1]. This highlights the importance of implementing pork classification based on desirable quality traits, such as darker lean colour, which can benefit specific demands in both the domestic and international markets [2,3]; however, there is a need to improve current lean colour measurement methods. In industry applications, meat colour measurements are often performed by experienced graders by assessing fresh pork surface colour against a subjective colour score standard, which is susceptible to inconsistency of scores among graders [4]. Current spectrophotometer and colourimeter technologies, such as the Minolta (Chiyoda City, Tokyo, Japan) and HunterLab (Reston, VA, USA) instruments are often used to produce colour coordinates (L*, a*, and b*) of meat samples defined by the Commission Internationale de l’Eclairage [5]. As up to 60% of journal articles use Minolta measurements to assess meat colour [6], and the NPPC [7] assigns Minolta L* values to accompany colour standards rather than HunterLab L* values [4], the Minolta is known to be used as a ‘*gold standard*’ assessment. Other factors also influence fresh meat colour measurement results, including light source (illuminant) and observer angle, with D_65_ illuminant at a 10° observer angle being the most used conditions to assess meat colour [6].

Multiple limitations exist when using traditional colourimeters to assess fresh lean colour in a commercial abattoir, including high cost, large size, and required operator training, which has restricted their large-scale application in industrial settings. Smaller and lower cost instruments such as Nix sensors (Nix Sensor Ltd., Hamilton, ON, Canada) and Spectro 1 and Color Muse series sensors (Variable Inc., Chattanooga, TN, USA) have attracted attention from commercial processing facilities. These sensors are designed for cost-effective and accurate paint colour assessment in the interior design industry; however, recent studies have demonstrated the potential use of the first model of the Nix Pro instrument to measure meat colour for beef [8,9,10]. Preliminary results in pork have revealed that other versions of these devices, such as the Nix Mini (Nix Sensor Ltd., Hamilton, ON, Canada), have lower correlations with traditional colourimeters than the newer models, Nix Pro II and Nix QC [11]. Other studies have used the Color Muse colourimeter, and revealed higher a* and b* values of meat samples from various species when compared to Minolta instruments [12].

Currently, the Spectro 1 and Spectro 1 Pro instruments, two different models of a compact spectrophotometer both produced by Variable Inc., have not been tested as an instrument for colour measurements in meat. These four hand-held colour sensors may be used to evaluate fresh pork colour with precise and reliable results. However, the correlation of the measurements from these novel instruments and those from traditional instruments and commercial subjective standards remains unconfirmed. Thus, the aim of this study was to evaluate the performance of Nix and Spectro 1 series instruments (Nix Pro II and Nix QC; Spectro 1 and Spectro 1 Pro) on assessing fresh pork colour, compared to the Minolta spectrophotometer and subjective colour scoring standards.

## 2. Materials and Methods

### 2.1. Measuring Canadian Pork Colour Standards

Instrumental colour was measured using two Minolta CM-700 spectrophotometers, four Nix sensors (two Nix Pro II and two Nix QC), and four Spectro 1 instruments (two Spectro 1 and two Spectro 1 Pro). The aperture size of Minolta, Spectro 1, and Spectro 1 Pro instruments is 8 mm. The Nix Pro II and Nix QC sensors have an aperture size of 14 mm. All instruments were set to D_65_ illuminant at 10° observer angle. However, the Nix Pro II device uses native D_50_ illuminant at 2° observer angle, which are then mathematically converted by the instrument to D_65_ at 10°. The Canadian Pork Colour Standard system [13] is a subjective scoring method used to evaluate fresh pork colour, and is represented by a visual chart with seven different colours representing seven different scores (range 0–6, 0 = extremely pale colour and 6 = extremely dark red colour). The standard score card was printed with opaque and colourfast ink on white plastic sticker material, which was then adhered to 2 mm thick opaque white plastic board. Standards were placed on the black surface benchtop. To evaluate the performance of the instruments in homogeneous and repeatable samples, the Canadian pork colour standards were measured using each instrument and model. Each of the seven colour scores were scanned ten consecutive times per day, on three separate days, using each instrument.

### 2.2. Instrumental Measurements of Commercial Pork

Fresh retail pork samples representing a wide variety of colour were purchased from five different local supermarkets. All samples were overwrapped with plastic food film in Styrofoam trays, and included 252 loin chops (*longissimus* muscle), and 46 tenderloins (*psoas major* muscle). Tenderloin samples were included due to the lack of commercial loin chops representing the darkest colours in the subjective scoring system. The average thickness of both loin chops and tenderloins was 2.5 mm. Once the packages were opened, all fresh retail pork samples underwent the same chilling (stored 4 °C for 24 h), handling, and measurement conditions. The cut surface of each loin chop and tenderloin was measured at eight and four evenly divided areas, respectively, using the same instruments as previously described. Meat samples were also subjectively evaluated by a trained grader using both the Canadian [13] and Japanese pork colour standard cube [14]. Only whole scores were assigned. The Japanese colour scores range from 1–6 (1 = extremely pale colour and 6 = extremely dark red colour). The Japanese colour standards were included to represent the system widely used in international markets.

### 2.3. Statistical Analysis

Colour measurement data on Canadian colour standards and fresh pork were analysed using PROC MIXED (SAS 9.4, SAS Institute Inc., Cary, NC, USA), with instrument model as fixed factor. The order of each instrument was randomized. The Bland-Altman plot [15] was also used to compare the Minolta and Nix series or Spectro 1 series instruments on measuring Canadian colour standards and pork surface colour. The Bland-Altman plot provides the mean difference (mean) between two methods of measurement (bias), and the standard deviation of the differences (SD) represents the random oscillations around the mean. The 95% limits of agreement were calculated for each colour coordinate based on ±1.96 SD (~2 SD) from the comparison of Minolta and the four handheld instruments. For the values recorded from the colour standards, relative standard deviation (RSD) for each instrument was calculated within the same day (intra-day), and among three different days (inter-day), as well as between two models of the same instrument (intra-model). Fresh pork data were analysed as “Whole muscle”, including all measurements from one sample, and “Central area”, including only the four central measurements. Correlations on the average value from fresh pork between each instrument and Minolta, as well as correlations between instrumental measurements from all instruments and subjective scores, were calculated using PROC CORR of SAS 9.4.

## 3. Results and Discussion

### 3.1. Instrumental Comparison on Canadian Colour Standard

From score 0 to score 6, the Spectro 1 Pro instruments had the highest L*, and the Spectro 1 instruments showed the lowest L* value (Table 1). The difference between the lowest and the highest value ranged from 2 to 3 units, which indicated the lightness value from Spectro 1 Pro was higher than Spectro 1. For a*, the Nix Pro II instruments displayed the highest values from score 0 to 6. The Spectro 1 instrument had the lowest a* value from score 0 to 3, and the Spectro 1 Pro instrument had the lowest a* value from score 4 to 6. For b* value, the Spectro 1 instruments had the highest values for score 0 and score 1 but had the lowest value from score 4 to score 6. For score 3, the Minolta instruments presented the highest b* value of 11.41, and the Spectro 1 Pro instruments had the lowest b* value of 8.51. From score 4 to 6, the Nix Pro II instruments produced the highest b* value. The major differences observed among instrument models may be due to their technical specifications. The Nix Pro II instruments use a different native illuminant (D_50_, 2°), with the illuminant D_65_ 10° setting being mathematically converted. Spectrophotometers, such as the Minolta CM 700 and the Spectro 1 instruments, use reflected light to quantify energy across the light spectrum. The reflected energy amount is then converted to colour value. Colourimeters, such as the Nix instruments, use a detector to measure the energy which passes through the filter (between the light source and sample) and reflects from the sample surface [16]. Additionally, each instrument uses different algorithms to calculate colour value, which may not indicate the changes on Canadian pork colour standard in the same degree.

**Figure 1:**
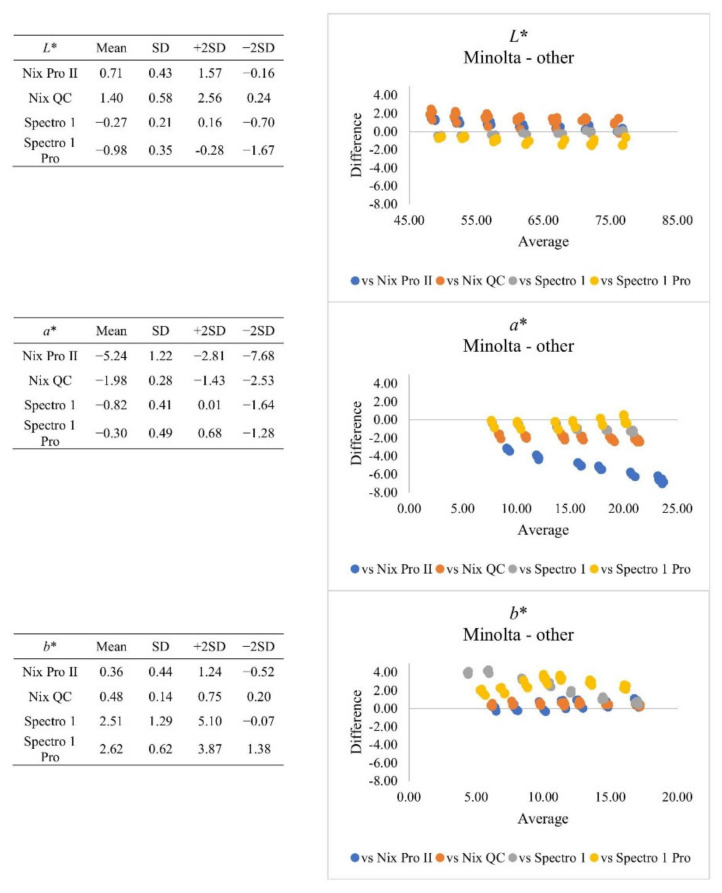
Plot of differences between Minolta and other instruments when Canadian colour standards were measured. SD = standard deviation.

In addition to descriptive statistics of colour measurements, the Bland-Altman plot (Figure 1) revealed that for L* value, both Nix and Spectro series instruments presented relatively less bias (~1), and the range of agreement was narrower, compared to a* and b* value. For a* value, the Nix Pro II had the greater bias (−5.24), compared to other instruments, and this bias was negative. For b* value, the Nix series instruments presented less bias (0.36–0.48), compared to Spectro 1 series (2.51–2.62). When b* value ranged from 5–10, Nix series instruments tended to have greater bias to Minolta measurements compared to the Spectro 1 series. The mean comparison results also indicated that the instruments from the same manufacturer produced similar bias (Nix vs. Spectro 1). The only exception was for the a* value generated by Nix Pro II. These differences in colour measurements, especially for a* and b* value, may be due to the different calculation mechanisms of reflected surface light. In addition, the Nix Pro II device uses native D_50_ illuminant at a 2° observer angle, which is then mathematically converted by the instrument to D_65_ at 10° [17,18]. While the Spectro 1 instruments are designed to obtain different values from shiny and matte surfaces (“visual colour”), the Spectro 1 Pro instruments account for this difference and provide the same results for both surfaces (“actual colour”). Overall, all instruments tended to generate equal increments or decrements for lower colour scores.

The RSD results of intra-day analysis were all under 5%, and ranged from 0.01 to 0.03%, 0.05 to 0.18%, and 0.08 to 0.46%, for L*, a*, b* trait, separately (Table 2). For inter-day analysis, RSD results for L*, a*, b* were all under 1%, except for b* from the Nix Pro II instruments (1.11%) and the Spectro 1 Pro instruments (1.59%). These RSD values ranged from 0.07 to 0.48%, 0.21 to 0.98%, and 0.50 to 1.59%, for L*, a*, b* traits, respectively. RSD results of intra-model ranged from 0.21 to 0.62%, 0.41 to 2.35%, and 0.53 to 2.84%, for L*, a*, b*, respectively. Among all instruments, the Minolta and the Nix QC were the only two instruments with all RSD values below 1%. According to the manufacturers, the colour difference *(*∆E00) value of short-term measurement and inter-instrument agreement for the Spectro 1 series instruments are 0.05, and 0.2–0.5, respectively [17], and for the Nix series instruments are 0.1, and 0.30–0.75, respectively [18]. Apart from these product specifications, one Spectro 1 Pro instrument had unexpected calibration issues during the measurement process, which may have slightly altered the RSD results. Typically, accepted RSD values are lower than 5%, and the observed low RSD values in the current study suggest the data had low variability [19]. Although all instruments had RSD values < 5%, some instruments had lower values (<2%), which would be beneficial if implementing an instrument for colour measurement with high accuracy, repeatability and reproducibility in systems that require greater precision, such as research environments or quality-based export markets with low levels of acceptable sorting error.

### 3.2. Instrumental Comparison on Retail Meat Samples

The average L* values of fresh pork samples from the five instrument models ranged from 44 to 55, and the Nix and Spectro 1 instruments were significantly different (*p* < 0.05) from the Minolta instruments (Table 3). Minolta instruments presented the highest L* values, and Nix Pro II instruments presented the lowest L* values. The average a* values ranged from 4.3 (Spectro 1 Pro) to 7.7 (Nix Pro II), and the Spectro 1 instruments were not significantly different (*p* > 0.05) from Spectro 1 Pro instruments. The average b* values ranged from 8.1 (Nix Pro II) to 13.5 (Minolta). Nix Pro II instruments were not significantly different from Nix QC instruments, and Spectro 1 instruments were not significantly different from Spectro 1 Pro instruments (*p* > 0.05).

Although instruments generated significantly different L* coordinate values when compared with Canadian colour standard scores (Table 1), the difference between maximum and minimum values were lower than the differences observed on meat samples. This may be due to the different aperture diameter of each instrument and, therefore, the percentage of meat surface being measured. The larger aperture size could also infer a comparatively greater susceptibility to edge-losses, indicating light on the meat surface would be not reflected, and was considered absorbed (Holman et al., 2015). In turn, the reflectance would interfere with L* (lightness). Meanwhile, meat surface is not homogeneous, unlike the surface of a Canadian colour standard. The presence of two-tone colours, connective tissues, and intramuscular fat on the meat surface may result in inconsistent measurement results [20,21]. Particular to the current study, darker tenderloins were also mixed and measured by all instruments, which could lead to the larger variance on colour coordinates.

For whole muscle measurements, a strong correlation (|r| = 0.8 to 0.9) was observed for the average L* and a* value for the Nix and Spectro 1 instruments with the Minolta instruments (Table 4). For b* value, the correlation between Nix and Minolta instruments was close to 0.7, > 0.8 for the Spectro 1 instruments, but lower for the Spectro 1 Pro instruments (|r| = 0.32). Similar results were observed for central area measurements, indicating that a narrower surface colour range (central area), and the number of measurements had very limited effect on correlation results. Additionally, the lack of effect between whole muscles and central areas could be due to the central areas being adequately representative of variability in the muscles in this study. In theory, more replicates or measurements would decrease the standard error of mean [19]. In practice, based on the size of surface area, replicates of measurement could vary from 3 to 30 [6]. In a previous study, seven measurements could contribute to minimize the SEM (standard error of predicted mean) value when assessing beef colour via Nix Pro instruments [8]. In the current study, correlation coefficients of novel instruments to Minolta instruments did not present differently between four and eight measurements.

**Figure 2:**
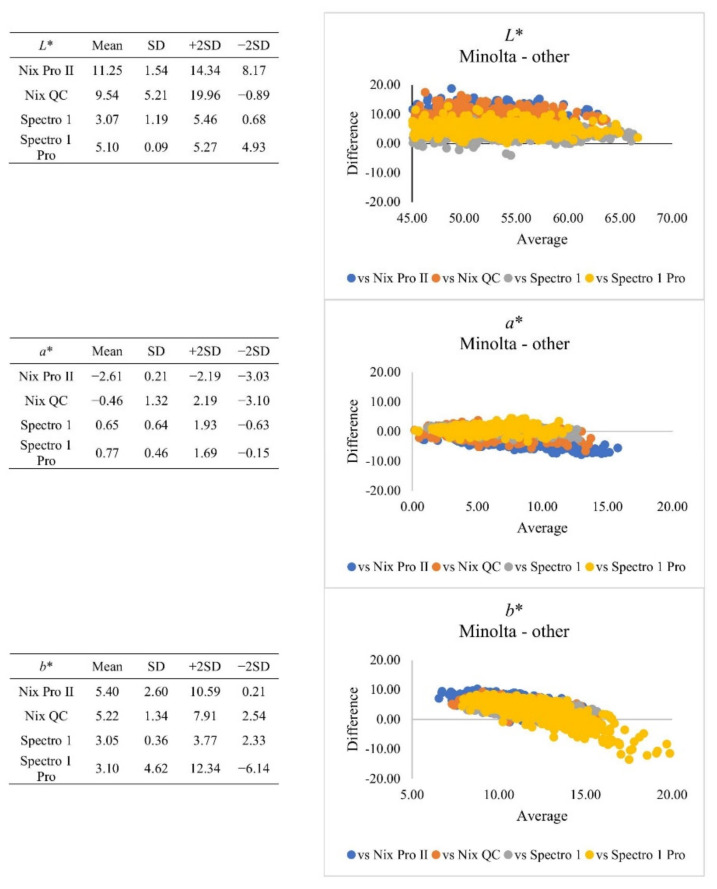
Plot of differences between Minolta and other instruments when retail meat samples were measured from whole muscles. SD = standard deviation.

**Figure 3:**
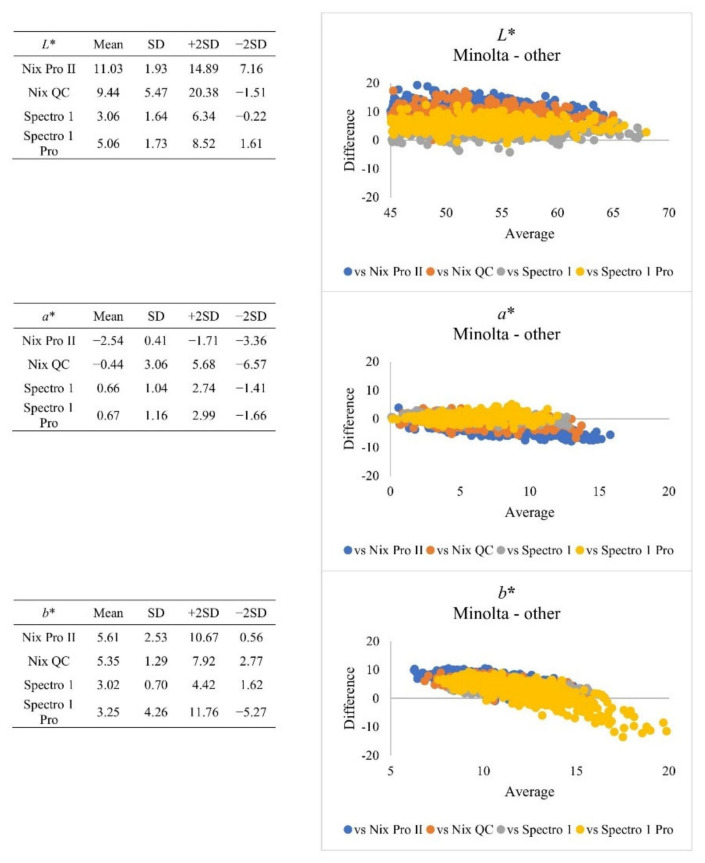
Plot of differences between Minolta and other instruments when retail meat samples were measured from central areas. SD = standard deviation.

Correlation is a statistical approach for determining if and how closely two variables are related. However, a strong correlation does not inherently suggest that the two methodologies are in accord [22]. The Bland-Altman plot (Figure 2) based on whole muscles revealed that for L* value, Spectro 1 series instruments presented relatively less bias (3.07–5.10), and the range of agreement was narrower, compared to Nix series instruments (bias: 9.54–11.25). The plot of differences from central areas presented similar results (Figure 3). But all four hand-held instruments presented significant (*p* < 0.05) strong correlation (|r| > 0.8) to Minolta. At the L* value range of 60–70, Spectro 1 and Nix instruments had lower bias when compared to Minolta. For a* value, the Nix Pro II had the greater bias (−2.61), compared to other instruments. When b* value ranged from 5–15, all instruments tended to have less bias to Minolta measurements. However, Spectro 1 Pro presented greater agreement bias, when b* value ranged from 10–20, when compared to the rest of recently developed instruments. The calibration protocols might also alter the measurements. Minolta, Nix QC, and Spectro 1 series instruments use calibration tiles, while Nix Pro II instruments use built-in software calibration. Different calibration techniques may lead to inconsistencies of colour coordinates [9]. At this point, it may not be conclusive to determine why these instruments produced different colour coordinates for pork samples in the current study. As the benchmark classification instrument, Minolta instruments have often been used to estimate standard colourimetric thresholds. The difference in absolute number value of colour coordinates from all instruments must be taken into account to avoid false positive or negative results before establishing alternative colour thresholds [10]. The colour acceptability threshold equated to the corresponding colour coordinate should be adjusted, depending on which specific instrument was used.

The measuring mechanism of the surface colour might impact the determination of b* value. As discussed in the previous section, Spectro 1 Pro instruments account for the shine of a surface. This feature may have had a large impact on the correlation of b* value to the Minolta instruments. It might have been more important to investigate L* and a* values when instrumental meat colour was measured, as the actual meat colour was a combination of L*, a*, and b* values. The higher correlation of the L* and a* value between novel instruments and Minolta, compared to correlations of b* values, may suggest that the L* and a* value may assess the lightness and red colour of fresh meat as b* values express the colour in the yellow-blue region of the spectrum, and the actual meat colour may present less colour variability in the yellow-blue region [23]. Additionally, while the Minolta was equipped with a xenon lamp as the light source, the Nix and Spectro 1 instruments use LED lamps. The different types of light sources displayed different emission spectra, which would affect how the colour was subjectively perceived. Red colour generally ranges from 600 to 700 nm in wavelength, and LED light displayed lower relative intensity (%) than xenon light in this region, which could result in different colour readings from instruments [24].

Since fat deposits and water residue on the surface may alter the instrument measurement of surface colour b* value [9], instruments designed to account for or ignore these differences would provide different results for this variable. In this study, the marbling content in tenderloin samples was negligible. Although heavy marbling regions on loin chops were excluded before dividing the meat surface into different locations for measurement, the trace amount of marbling might not be completely avoided, which would result in different number values on colour coordinates from different instruments. The strong correlation and mean difference comparison of novel instruments on colour coordinates (except b* from the Spectro 1 pro instrument) to the Minolta instruments revealed the potential of these instruments in developing alternative pork classification systems based on colour traits.

### 3.3. Instrumental Measurements and Subjective Standards

For whole muscle measurements, the correlation coefficients (|r|) between Canadian subjective standards and L* values from Nix instruments were >0.8 and were close to 0.8 from Spectro 1 series instruments and the |r| from Minolta spectrophotometer was <0.7 (Table 5). The correlation between Canadian subjective standards and a* value from Nix instruments and Spectro 1 instruments ranged from 0.6–0.7. Minolta instruments presented lower correlation (|r| = 0.47). Correlation between Canadian subjective standards and b* value from Nix instruments and Spectro 1 instruments was between ~0.3–0.4, and Minolta instruments had a lower correlation (|r| = 0.14).

For the central area measurements, the results were similar to whole muscle measurements (Table 5), with the exception that Minolta had increased correlations (|r|) on central area measurements for L* (0.68–0.76) and a* values (0.47–0.59). Similarly, the correlation of all instruments to the Japanese subjective standard were very similar (Table 6) to those with the Canadian subjective standards. The current study of Minolta instrument correlations presented stronger coefficients for L* and a* values, but very poor coefficients for b* value. Interestingly, for b* value, Spectro 1 instruments presented negative correlations, but Spectro 1 Pro instruments presented positive correlations. As discussed, the difference may be due to the instrumental calculation of the surface shine. The Canadian colour standard is divided into seven colour scores, which vary from pale pink (score 0) to dark red (score 6). Similarly, Japanese colour standards are divided into six colour scores. The visual appraisal from human eyes may not be indicative of different colour coordinates, as the subjective red colour in meat is a combination of L*, a*, and b* values [23]. The b* values had a small range when compared to the ranges in the L* and a* values (Table 1), which could have had less correlation to human score assignments.

In general, visual colour assessments are performed by trained personnel, and despite regular calibration and proper training, human errors are difficult to avoid [25]. However, the above hand-held instruments are also frequently operated by humans. In order to minimize human error, there is a need to study alternative innovative colour assessment instruments that can be automated. Although small hand-held devices like those evaluated in this study present potential for automation due to their compact size and connectivity capabilities, current versions may present difficulties for adaptation to in-plant use. For example, Spectro 1 series instruments automatically initiate self-correction procedures after approximately 10 measurements, which increase the single scanning time to up to 60 s. This feature guarantees the adequate performance of the instrument but presents limitations to their efficiency in meat processing lines, where an efficient scanning time is critical to meet processing speed. Another future application for these hand-held devices may be the establishment of acceptability thresholds for fresh and displayed meat for various settings, such as wholesale stores and retail stores. Furthermore, the importance of accurate and efficient assessment of a* values is emphasized, due to its association with fresh colour stability during storage [26,27]. Additionally, other important pork quality traits, such as pH and drip loss, may also correlate with instrumental colour measurement, thereby highlighting the need for a more comprehensive pork quality assessment system that could be established in the future.

## 4. Conclusions

This study explored the potential of Nix and Spectro 1 instruments as novel methods to measure fresh pork colour. Although the latter instruments presented strong correlations with the Minolta CM-700 spectrophotometer colour coordinates, the absolute values of the colour traits varied by instrument. Instrumental measurement from all devices had weak correlations for b* values between instrumental measure and Canadian subjective colour standard measure. The new hand-held instruments have the potential to become an alternate to Minolta due the affordability and potential for automation, especially for small-scale meat packers. All four hand-held instruments support cellphone Bluetooth connectivity, which ensure the convenience to export data for analysis. Moreover, due to the limited space in commercial processing facilities, the potential to develop the automated colour classification system with pocket-size instruments will be meaningful for the meat industry. However, specific technical specifications need to be considered before these devices can be implemented for the meat industry, such as the long calibration time required by the Spectro 1 series instruments after a number of scans.

## Figures and Tables

**Table 1:** Instrument effect on mean CIE (L*, a*, and b*) values of Canadian colour standards.

	Subjective Colour Score	Minolta	Nix Pro II	Nix QC	Spectro 1	Spectro 1 Pro	SEM
L*	0	76.78 ^b^	76.33 ^c^	75.51 ^d^	75.28 ^e^	77.57 ^a^	0.06
	1	71.88 ^b^	71.35 ^c^	70.57 ^d^	70.04 ^e^	72.93 ^a^	0.05
	2	67.49 ^b^	66.94 ^c^	66.20 ^d^	65.55 ^e^	68.55 ^a^	0.04
	3	62.13 ^b^	61.43 ^c^	60.75 ^d^	60.16 ^e^	63.21 ^a^	0.04
	4	57.40 ^b^	56.43 ^c^	55.81 ^d^	55.25 ^e^	58.24 ^a^	0.04
	5	52.81 ^b^	51.61 ^c^	50.96 ^d^	50.66 ^e^	53.34 ^a^	0.04
	6	49.31 ^b^	47.88 ^c^	47.27 ^d^	47.17 ^e^	49.88 ^a^	0.04
a*	0	7.95 ^d^	10.78 ^a^	9.23 ^b^	7.71 ^e^	8.02 ^c^	0.02
	1	11.04 ^c^	14.00 ^a^	11.77 ^b^	9.71 ^e^	10.53 ^d^	0.02
	2	15.10 ^c^	18.31 ^a^	15.31 ^b^	13.34 ^e^	14.04 ^d^	0.03
	3	17.07 ^b^	20.42 ^a^	17.12 ^b^	15.44 ^d^	15.62 ^c^	0.02
	4	20.11 ^b^	23.90 ^a^	19.93 ^c^	18.65 ^d^	18.00 ^e^	0.03
	5	22.73 ^b^	26.68 ^a^	22.31 ^c^	21.34 ^d^	20.04 ^e^	0.03
	6	22.70 ^b^	26.80 ^a^	22.38 ^c^	21.41 ^d^	20.05 ^e^	0.03
b*	0	16.97 ^b^	16.62 ^d^	16.88 ^c^	17.20 ^a^	14.90 ^e^	0.02
	1	14.56 ^ab^	14.37 ^c^	14.50 ^b^	14.60 ^a^	12.10 ^d^	0.02
	2	12.59 ^a^	12.40 ^b^	12.41 ^b^	11.83 ^c^	9.65 ^d^	0.03
	3	11.41 ^a^	11.31 ^b^	11.24 ^b^	9.80 ^c^	8.51 ^d^	0.02
	4	9.75 ^b^	9.84 ^a^	9.54 ^c^	7.05 ^e^	7.30 ^d^	0.03
	5	7.76 ^b^	7.91 ^a^	7.48 ^c^	3.96 ^e^	6.00 ^d^	0.03
	6	6.19 ^b^	6.35 ^a^	5.96 ^c^	2.66 ^e^	4.55 ^d^	0.03

Means within the same row with different superscripts were significantly different (*p* < 0.05).

**Table 2:** Variability of instrument measurements of Canadian pork colour standards represented by average relative standard deviation (RSD %).

RSD %	Colour	Instrument Model				
	Coordinate	Minolta	Nix Pro II	Nix QC	Spectro 1	Spectro 1 Pro
Intra-day ^i^	L*	0.01	0.02	0.01	0.03	0.03
	a*	0.05	0.13	0.14	0.13	0.18
	b*	0.08	0.12	0.11	0.41	0.46
Inter-day ^ii^	L*	0.07	0.16	0.47	0.48	0.09
	a*	0.21	0.55	0.98	0.31	0.33
	b*	0.50	1.11	0.86	0.69	1.59
Intra-model ^iii^	L*	0.50	0.45	0.36	0.62	0.21
	a*	0.56	1.03	0.93	0.41	2.35
	b*	0.53	2.69	0.75	1.93	2.84

^i^ Intra-day: measurements performed in same day. ^ii^ Inter-day: measurements performed over 3 consecutive days. ^iii^ Intra-model: measurements performed between two models of the same instrument.

**Table 3:** Descriptive statistics of mean colour coordinate value of retail meat samples for each instrument and model.

	Minolta	Nix Pro II	Nix QC	Spectro 1	Spectro 1 Pro
L*	55.07 ^a^ ± 5.20	43.83 ^e^ ± 5.83	45.69 ^d^ ± 5.68	52.00 ^b^ ± 5.12	49.97 ^c^ ± 5.33
a*	5.05 ^c^ ± 2.42	7.66 ^a^ ± 3.35	5.48 ^b^ ± 2.52	4.40 ^d^ ± 2.72	4.27 ^d^ ± 2.01
b*	13.53 ^a^ ± 1.29	8.13 ^c^ ± 2.29	8.19 ^c^ ± 1.94	10.48 ^b^ ± 1.38	10.43 ^b^ ± 3.36

Means within the same row with different superscripts were significantly different (*p* < 0.05).

**Table 4:** Correlations between meat colour measurements from Minolta and novel instruments.

		Nix Pro II	Nix QC	Spectro 1	Spectro 1 Pro
Whole Muscle	L*	0.82	0.84	0.92	0.90
	a*	0.82	0.86	0.93	0.88
	b*	0.68	0.68	0.76	0.32
Central Area	L*	0.85	0.87	0.93	0.92
	a*	0.86	0.89	0.95	0.89
	b*	0.68	0.69	0.78	0.30

All correlation coefficients were significantly different (*p* < 0.05).

**Table 5:** Correlation coefficients of instrumental measurement with Canadian pork colour standard on fresh pork colour.

		Minolta	Nix Pro II	Nix QC	Spectro 1	Spectro 1 Pro
Whole Muscle	L*	−0.68	−0.85	−0.85	−0.80	−0.78
	a*	0.47	0.69	0.66	0.67	0.60
	b*	−0.14	0.31	0.32	−0.39	0.41
Central Area	L*	−0.76	−0.86	−0.84	−0.80	−0.78
	a*	0.59	0.69	0.64	0.67	0.59
	b*	−0.13	0.31	0.31	−0.35	0.42

All correlation coefficients (r) were significantly different (*p* < 0.05).

**Table 6:** Correlation coefficients of instrumental measurement with Japanese pork colour standard on fresh pork colour.

		Minolta	Nix Pro II	Nix QC	Spectro 1	Spectro 1 Pro
Whole Muscle	^1^ L*	−0.68	−0.83	−0.84	−0.79	−0.77
	a*	0.47	0.68	0.64	0.65	0.58
	b*	−0.13	0.25	0.29	−0.39	0.41
Central Area	L*	−0.75	−0.83	−0.83	−0.78	−0.76
	a*	0.58	0.67	0.63	0.65	0.57
	b*	−0.12	0.26	0.29	−0.35	0.41

^1^ All correlation coefficients (r) were significantly different (*p* < 0.05).

## Data Availability

Datasets generated during the current study are available from the corresponding author on reasonable request.

## References

[1] King D.A., Shackelford S.D., Wheeler T.L. (2011). Use of visible and near-infrared spectroscopy to predict pork longissimus lean color stability. J. Anim. Sci..

[2] Ngapo T.M. (2017). Consumer preferences for pork chops in five Canadian provinces. Meat Sci..

[3] Ngapo T.M., Martin J.F., Dransfield E. (2007). International preferences for pork appearance: II. Factors influencing consumer choice. Food Qual. Prefer..

[4] Bohrer B.M., Boler D.D. (2017). REVIEW: Subjective pork quality evaluation may not be indicative of instrumental pork quality measurements on a study-to-study basis. Prof. Anim. Sci..

[5] CIE (1978). Recommendations on Uniform Color Spaces—Color-Difference Equations, Psychometric Color Terms = Recommandations Sur Les Espaces Chromatiques Uniformes—Les Formules de Difference de Couleur, Les Termes Psychometriques de la Couleur.

[6] Tapp W.N., Yancey J.W.S., Apple J.K. (2011). How is the instrumental color of meat measured?. Meat Sci..

[7] NPPC (1999). Official Color and Marbling Standards.

[8] Holman B.W.B., Collins D., Kilgannon A.K., Hopkins D.L. (2018). The effect of technical replicate (repeats) on Nix Pro Color Sensor™ measurement precision for meat: A case-study on aged beef colour stability. Meat Sci..

[9] Holman B.W.B., Hopkins D.L. (2019). A comparison of the Nix Colour Sensor Pro™ and HunterLab MiniScan™ colorimetric instruments when assessing aged beef colour stability over 72 h display. Meat Sci..

[10] Holman B.W.B., Kerr M.J., Morris S., Hopkins D.L. (2019). The identification of dark cutting beef carcasses in Australia, using Nix Pro Color Sensor™ colour measures, and their relationship to bolar blade, striploin and topside quality traits. Meat Sci..

[11] Wei X., Bohrer B., Lopez-Campos O., Prieto N., Uttaro B., Juarez M. Comparing Nix sensor and Minolta colourimeter to measure instrumental colour in fresh pork. Proceedings of the Banff Pork Seminar, University of Alberta.

[12] Dang D.S., Buhler J.F., Stafford C.D., Keele N.E., Esco A.N., Yang J., Matarneh S. Color muse colorimeter as an alternative method for measuring color in meat. Proceedings of the 73rd Reciprocal Meat Conference.

[13] Maignel L., Fortier M.P., Lambert P., Riendeau L., Wyss S., Sullivan B. Defining carcass and meat quality standards for Canadian pork: Meat colour. Proceedings of the 58th International Congress of Meat Science and Technology.

[14] Nakai H., Saito F., Ikeda T., Ando S., Kamatsu A. (1975). Standard models of pork-colour. Bull. Natl. Inst. Anim. Ind..

[15] Bland J.M., Altman D. (1986). Statistical methods for assessing agreement between two methods of clinical measurement. Lancet.

[16] Brewer M.S., Novakofski J., Freise K. (2006). Instrumental evaluation of pH effects on ability of pork chops to bloom. Meat Sci..

[17] Variable Inc Introducing Spectro 1 Pro. https://variableinc.com/spectro-1-pro.html.

[18] Nix Sensor Ltd Nix Hardware Comparison. https://www.nixsensor.com/compare-nixes/.

[19] Mason R.L., Gunst R.F., Hess J.L. (2003). Statistical Design and Analysis of Experiments: With Applications to Engineering and Science.

[20] Girolami A., Napolitano F., Faraone D., Braghieri A. (2013). Measurement of meat color using a computer vision system. Meat Sci..

[21] Holman B.W.B., Ponnampalam E.N., van de Ven R.J., Kerr M.G., Hopkins D.L. (2015). Lamb meat colour values (HunterLab CIE and reflectance) are influenced by aperture size (5 mm v. 25 mm). Meat Sci..

[22] Giavarina D. (2015). Understanding Bland Altman analysis. Biochem. Med..

[23] Brewer M.S., Zhu L.G., Bidner B., Meisinger D.J., McKeith F.K. (2001). Measuring pork color: Effects of bloom time, muscle, pH and relationship to instrumental parameters. Meat Sci..

[24] Martin D. A Practical Guide to Machine Vision Lighting. https://www.advancedillumination.com/wp-content/uploads/2018/10/A-Practical-Guide-to-Machine-Vision-Lighting-v.-4-Generic.pdf.

[25] Goni V., Indurain G., Hernandez B., Beriain M.J. (2008). Measuring muscle color in beef using an instrumental method versus visual color scales. J. Muscle Foods.

[26] Khliji S., Van de Ven R., Lamb T., Lanza M., Hopkins D.J.M.S. (2010). Relationship between consumer ranking of lamb colour and objective measures of colour. Meat Sci..

[27] Holman B.W.B., van de Ven R.J., Mao Y., Coombs C.E.O., Hopkins D.L. (2017). Using instrumental (CIE and reflectance) measures to predict consumers’ acceptance of beef colour. Meat Sci..

